# High glucose alters tendon homeostasis through downregulation of the AMPK/Egr1 pathway

**DOI:** 10.1038/srep44199

**Published:** 2017-03-07

**Authors:** Yu-Fu Wu, Hsing-Kuo Wang, Hong-Wei Chang, Jingyu Sun, Jui-Sheng Sun, Yuan-Hung Chao

**Affiliations:** 1 School and Graduate Institute of Physical Therapy, College of Medicine, National Taiwan University, Taipei, Taiwan; 2 Center of Physical Therapy, National Taiwan University Hospital, Taipei, Taiwan; 3 Nephrology Division, Department of Internal Medicine, National Taiwan University Hospital, Taipei, Taiwan; 4 Sports and Health Research Center, Tongji University Department of Physical Education, Shanghai, China; 5 Department of Orthopedic Surgery, College of Medicine, National Taiwan University, Taipei, Taiwan; 6 Department of Orthopedic Surgery, National Taiwan University Hospital, Taipei, Taiwan; 7 Rehabilitation Center, National Taiwan University Hospital Chu-Tung Branch, Hsinchu County, Taiwan.

## Abstract

Diabetes mellitus (DM) is associated with higher risk of tendinopathy, which reduces tolerance to exercise and functional activities and affects lifestyle and glycemic control. Expression of tendon-related genes and matrix metabolism in tenocytes are essential for maintaining physiological functions of tendon. However, the molecular mechanisms involved in diabetic tendinopathy remain unclear. We hypothesized that high glucose (HG) alters the characteristics of tenocyte. Using *in vitro* 2-week culture of tenocytes, we found that expression of tendon-related genes, including Egr1, Mkx, TGF-β1, Col1a2, and Bgn, was significantly decreased in HG culture and that higher glucose consumption occurred. Down-regulation of Egr1 by siRNA decreased Scx, Mkx, TGF-β1, Col1a1, Col1a2, and Bgn expression. Blocking AMPK activation with Compound C reduced the expression of Egr1, Scx, TGF-β1, Col1a1, Col1a2, and Bgn in the low glucose condition. In addition, histological examination of tendons from diabetic mice displayed larger interfibrillar space and uneven glycoprotein deposition. Thus, we concluded that high glucose alters tendon homeostasis through downregulation of the AMPK/Egr1 pathway and the expression of downstream tendon-related genes in tenocytes. The findings render a molecular basis of the mechanism of diabetic tendinopathy and may help develop preventive and therapeutic strategies for the pathology.

People with diabetes mellitus (DM) are susceptible to tendinopathy and tendon rupture^1^,^2^. Diabetic tendons are characterized by weaker mechanical properties than non-DM tendons, including lower stiffness, maximum load, Young’s modulus, and strain at break point^3^. Tendons are mainly composed of highly organized collagen fibers as well as various minor structural molecules, such as proteoglycans and glycoproteins^4^. In tendons, tenocyte is the fundamental cell population responsible for maintaining tissue homeostasis, and loss of its genetic traits can cause tendon dysfunction^5^.

Several transcription factors have been identified as crucial genetic regulators of tendon development and repair, including Scleraxis (Scx), Mohawk (Mkx), and Early growth response factor 1 (Egr1). All of them were shown to regulate the expression of matrix molecule genes in developing tendons, including type 1 collagen and Tenomodulin (Tnmd)^6^,^7^,^8^,^9^,^10^. Scx is the best-studied teno-lineage marker and has been shown to be essential for the formation of tendons. Scx-null mutant mice tendons displayed poor organization in anchoring tendons whereas the force-transmitting tendons drastically failed or were even completely absent^11^,^12^. While Scx played a pivotal role in the initiation of tenocyte differentiation, Mkx and Egr1 were suggested to be involved in the maintenance of differentiated tenocytes^8^,^13^. Ablation of either Mkx or Egr1 led to reduction in diameter and tensile strength of tendons in mutant mice^8^,^10^. In addition, both Mkx and Egr1 directly promoted expression of TGF-βs^10^,^14^, whose signaling is critical in tendon development, and both were shown to be disrupted in diseased human tendons^15^,^16^,^17^. Although a previous study has demonstrated that oxidative stress upregulated Scx expression of tenocytes under a 5 mM-glucose condition, whereas it induced apoptosis under a 17.5 mM-glucose condition^18^, understanding of the influence of DM on Mkx and Egr1 expression in tendons is still lacking.

Glucose is the main energy source of all cell types. By utilizing glucose via metabolic processes, cells meet their needs for energy. AMP-activated protein kinase (AMPK) is an energy-sensor, which can be activated allosterically by adenosine monophosphate (AMP) and adenosine diphosphate (ADP) when the cellular energy state becomes low^19^. AMPK regulates a diverse array of cellular processes involved in diabetes, inflammation, and carcinogenesis such as cell metabolism, proliferation, differentiation, and apoptosis^19^,^20^. *In vitro* studies have revealed that cells from connective tissues were susceptible to extracellular glucose concentrations, resulting in decreased AMPK activity under high glucose conditions^21^,^22^. Hence, using primary rat tenocyte culture in the present study, we examined the hypothesis that a high glucose environment affects the expression of pivotal transcription factors as well as downstream tendon-associated molecules via inactivation of AMPK and proposed a pathomechanism underlying diabetic tendinopathy.

## Results

### No significant effect on cell growth and apoptosis

**Figure 1:**
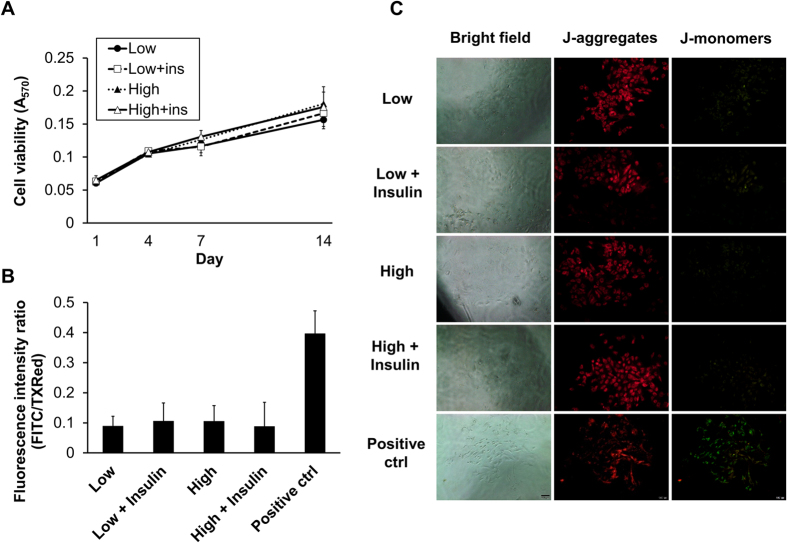
Effects of glucose and insulin on tenocyte growth and apoptosis. (**A**) Tenocyte viability was similar among all groups of high- and low-glucose concentration (HG: 4.5 g/L; LG: 1.0 g/L), with or without insulin, from day 1 to day 14 (n = 8). As shown (**B**) quantitatively and (**C**) micrographically by mitochondrial membrane potential analysis, no obvious signs of apoptosis were found in all of the four groups at day 14. The positive control group was treated with hydrogen peroxide for 16 hrs (n = 3).

Throughout the two-week culture, similar viability levels of tenocytes were observed in low- and high-glucose medium (LG and HG) with or without insulin at each time point (Fig. 1A). For each of the four groups, the results of the JC-1 assay showed no differences in mitochondrial membrane potentials over time, which would exhibit changes in an apoptosis process (Fig. 1B,C).

### Increased glucose uptake and consumption in the HG condition

**Figure 2:**
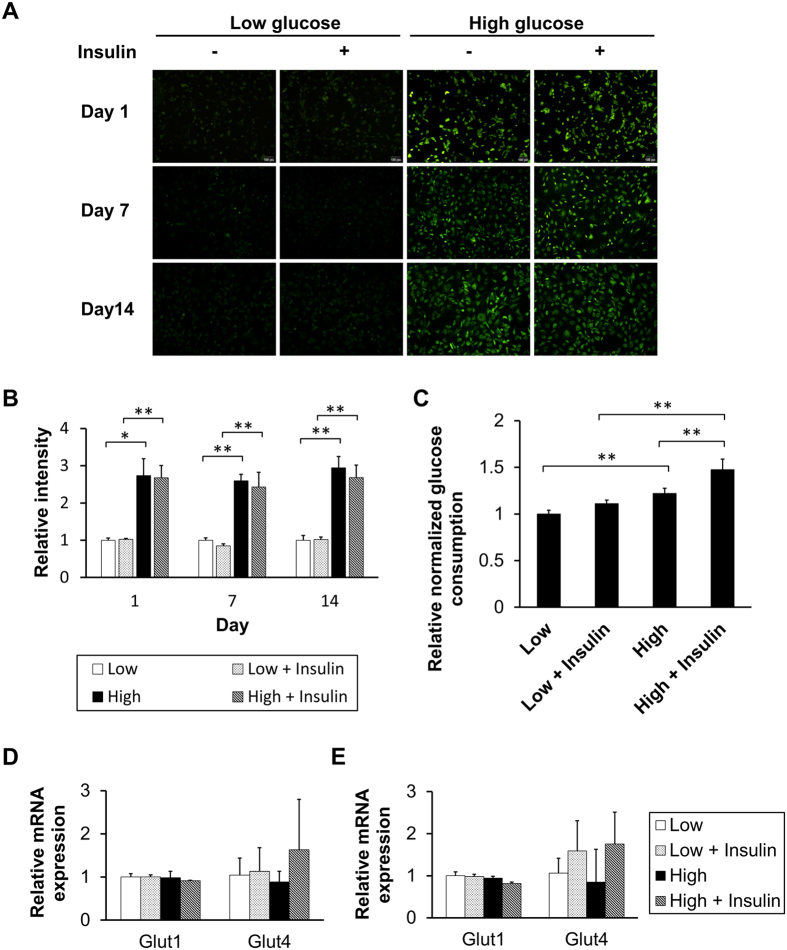
Effects of glucose and insulin on glucose uptake and consumption. As shown (**A**) micrographically and (**B**) quantitatively by 2-NBDG uptake analysis, glucose uptake was increased by increasing glucose level, but not by increasing insulin (n = 4). (**C**) Glucose consumption was raised by increasing glucose, and by increasing insulin (n = 4). mRNA levels of glucose transporters were not significantly different between the four groups, both at day 7 (**D**) and day 14 (**E**) (n = 6).

At day 1, 7, and 14, tenocytes in HG conditions had higher glucose uptake than those in LG conditions, while insulin did not affect the glucose uptake efficiency in either the HG or LG condition (Fig. 2A,B). We also demonstrated that tenocytes in the HG conditions consumed more glucose than those in the LG conditions. Insulin significantly increased the glucose consumption in the HG condition but not in the LG condition (Fig. 2C). The real-time PCR analysis showed that the expression of glucose transporters, Glut1 and Glut4, were not significantly altered in any of the groups at days 7 and 14 (Fig. 2D,E).

### HG altered tendon-related gene expression

**Figure 3:**
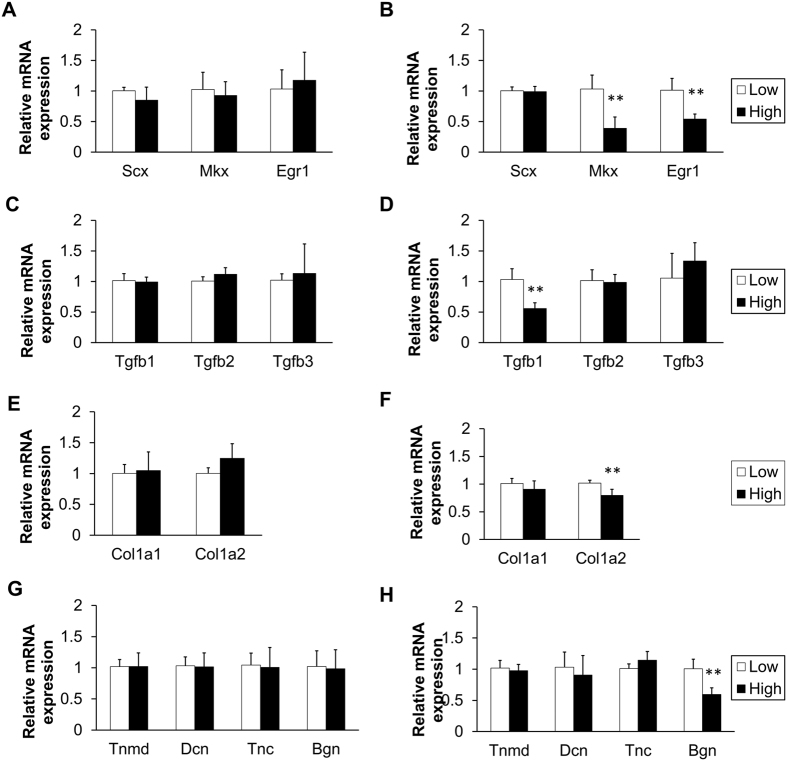
Effects of glucose conditions on tendon-related gene expression. (**A,C,E** and **G**) At day 7, no alteration was found in the expression of all the genes tested. (**B,D,**F and **H**) At day 14, the HG condition significantly attenuated mRNA expression of Mkx, Egr1, TGF-β1, Col1a2, and Bgn (n = 6; **p < 0.01 compared with the LG group).

Although they survived and grew equally well in the HG and LG conditions, tenocytes had higher glucose uptake and consumed more glucose in the HG condition. Thus, we analyzed the expression of tendon-associated genes in the tenocytes. First, we focused on the crucial transcription factors. At day 7, none of the transcription factors were changed by different glucose levels (Fig. 3A). However, Mkx and Egr1 expression were suppressed in the HG condition at day 14 (Fig. 3B). Specifically, the expression levels of Mkx and Egr1 in the HG group were 0.39 ± 0.18-times and 0.54 ± 0.08-times as high as the LG control group, respectively. Next, we examined downstream genes including TGF-βs and matrix molecules. While all of the genes remained unchanged at day 7 (Fig. 3C,E and G), we found that TGF-β1, Col1a2 and Biglycan (Bgn) were significantly downregulated in the HG condition at day 14 (Fig. 3D,F and H). Expression levels of these genes were 0.56 ± 0.09-times, 0.80 ± 0.11-times, and 0.60 ± 0.11-times as high as the LG group, respectively.

### Insulin altered tendon-related gene expression

**Figure 4:**
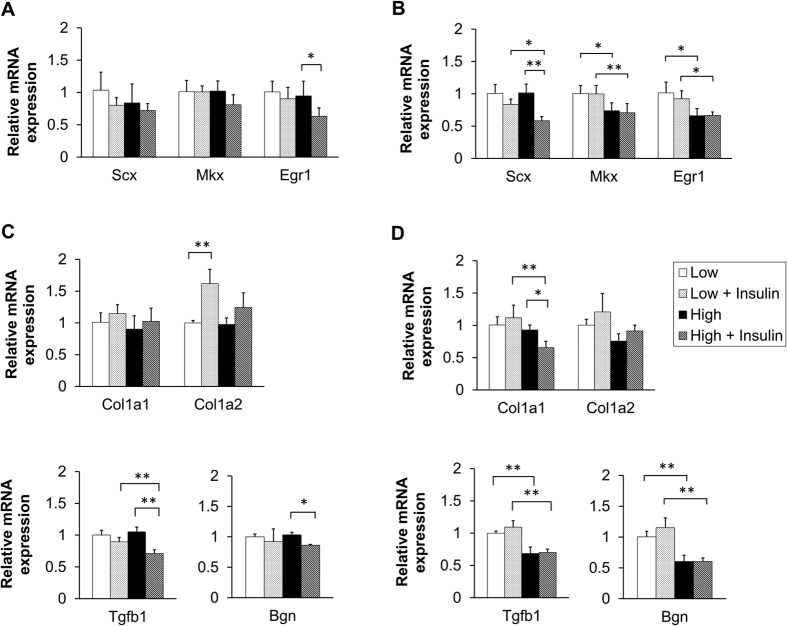
Effects glucose and insulin on tendon-related gene expression. (**A** and **C**) At day 7, mRNA expression of Egr1, TGF-β1, and Bgn were reduced by insulin treatment in the HG condition. (**B** and **D**) At day 14, while mRNA expression of Scx and Col1a1 were suppressed by insulin in the HG condition, no further effects were found on the expression of Mkx, Egr1, TGF-β1, and Bgn (n = 6; *p < 0.05, **p < 0.01).

We demonstrated that the glucose consumption in the HG condition with insulin was significantly higher than in the HG condition without insulin. To examine whether higher glucose consumption resulted in even lower tendon-related gene expression, we tested the expression of three crucial transcription factors, including Scx, Mkx, and Egrl. TGF-β1, Collagen 1, and Bgn genes were tested as well. At day 7, expression of Egr1, TGF-β1, and Bgn were suppressed by insulin under the HG condition, with levels ~67%, ~68%, and ~84% as high as the non-insulin HG group, respectively (Fig. 4A and C). At day 14, Scx and Col1a1 were significantly suppressed by insulin, to ~58% and ~70% the levels of the non-insulin HG group, respectively. Nevertheless, expression of Mkx, Egr1, TGF-β1, and Bgn were not further affected by insulin under the HG condition, while all of them were lower than in LG conditions (Fig. 4B and D). Therefore, we conclude that insulin also altered the genes which were regulated by glucose. However, this occurred at an early time point.

### Egr1 siRNA downregulated tendon-related gene expression

**Figure 5:**
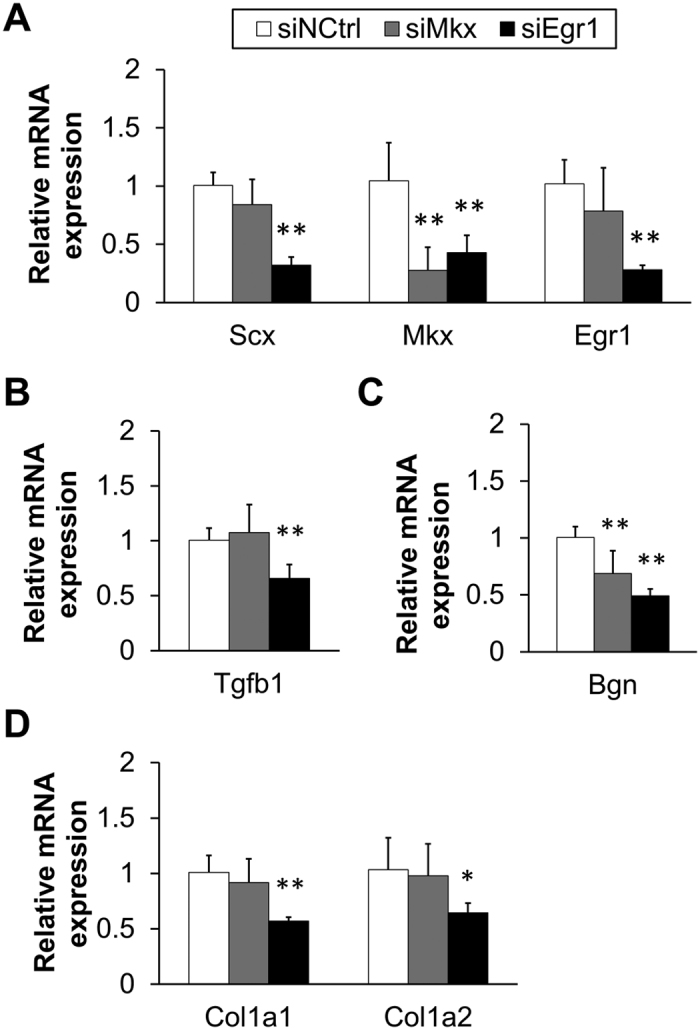
Effects of Egr1 and Mkx siRNA on downstream gene expression. Silencing of Egr1 expression by siRNA down-regulated the mRNA expression of (**A**) Scx and Mkx, (**B**) TGF-β1, (**C**) Bgn, (**D**) Cola1 and Col1a2. Bgn expression was also attenuated by siRNA-mediated silencing of Mkx (n = 6; *p < 0.05, **p < 0.01 compared with the negative control group).

To examine the hypothesis that observed changes in downstream genes under the different conditions were regulated by transcription factors, and to further investigate which transcription factor played a more important role, we used siRNA to knockdown the expression of Mkx or Egr1. We found that Bgn was the only gene that decreased when Mkx was knocked-down (Fig. 5C). However, after inhibiting Egr1 to a level of 0.28 ± 0.04-times as high as that of the control group, expression levels of all the target genes, including TGF-β1, Bgn, Col1a1, and Col1a2, were significantly suppressed (Fig. 5B–D). In addition, transcription factor Scx and Mkx were also down-regulated by the Egr1 suppression (Fig. 5A).

### AMPK-dependent regulation of tendon-related gene expression

**Figure 6:**
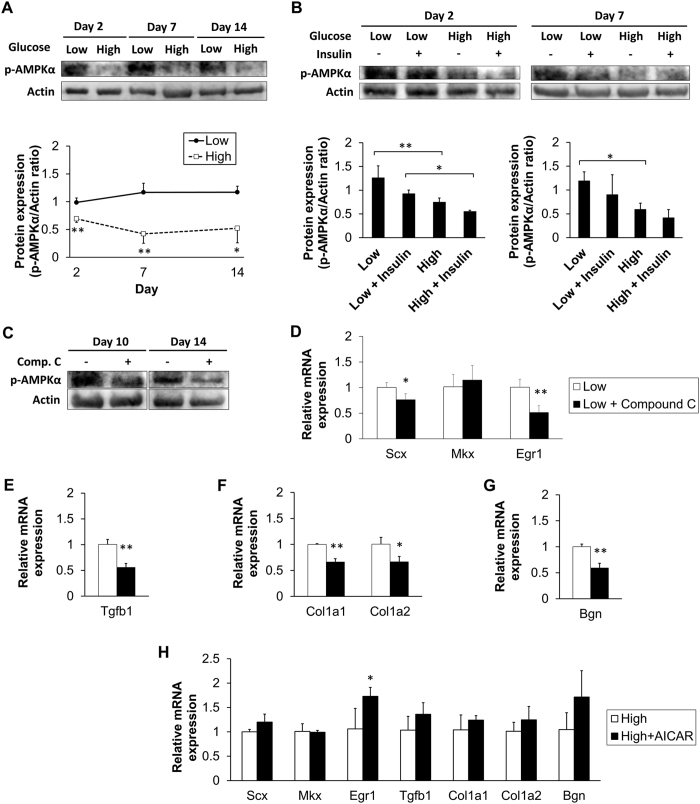
Inactivation of AMPK impaired the expression of tendon-related genes. (**A**) Western blotting showed lower protein expression levels of phospho-AMPK (p-AMPK) at day 2, 7, and 14 in the HG condition. β-actin was used as a loading control (n = 3). (**B**) Western blotting showed that insulin further decreased p-AMPK protein expression at day 2 and 7 (n = 3). (**C**) Treatment of compound C, an AMPK inhibitor, beginning from day 7 continuously inhibited p-AMPK expression. Compound C attenuated mRNA expression of (**D**) Scx, Egr1, (**E**) TGF-β1, (**F**) Collagen 1, and (**G**) Bgn (n = 4; *p < 0.05, **p < 0.01 compared with the untreated LG group). (**H**) Tenocytes grown in HG condition were treated with AICAR (0.2 mM), an AMPK activator. AICAR significantly increased Egr1 expression (n = 4; *p < 0.05 compared with the untreated HG group). Uncropped Western blot gels related to this figure are displayed in Supplementary Fig. S2.

We hypothesized that increased glucose consumption in tenocytes under HG conditions resulted in inactivation of AMPK signaling, which in turn affected the expression of tendon-related genes. To test our hypothesis, we performed western blotting analysis and found that p-AMPKα expressed less in the HG than in the LG condition at days 2, 7, and 14 (Fig. 6A). Insulin further reduced p-AMPKα expression in the HG condition to some extent, which was more significant at day 2 than at day 7 (Fig. 6B). To figure out the connection between AMPK inactivation and gene alteration, we treated the tenocytes with an AMPK inhibitor, Compound C, under the LG condition at day 7, the time point when HG inactivated AMPK the most. After the seven-day treatment, tenocytes expressed less p-AMPKα than the control group at day 14 (Fig. 6C). Using real-time PCR, we demonstrated that the expression level of Egr1 had reduced to 0.51 ± 0.13-times the level of the control group. Moreover, expression of the Egr1 downstream targets TGF-β1, Col1a1, Col1a2, and Bgn, as well as of Scx, was significantly decreased by AMPK inhibition, whereas Mkx was not affected (Fig. 6D–G). To determine if AMPK activation under HG conditions mitigates these changes, we treated tenocytes grown in HG condition with AICAR, an AMPK activator. The expression of the Egr1 was significantly increased by AMPK activation (Fig. 6H).

### Fiber disorganization and increased interfibrillar spaces in diabetic tendon

**Figure 7:**
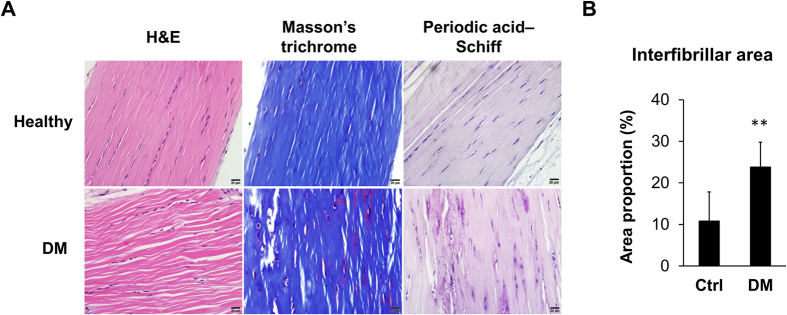
Tendons from STZ-induced type 1 DM mice (duration >1 yr) showed disorganization in ECM structure. (**A**) H&E, Masson’s trichrome, and PAS stained longitudinal sections of mice Achilles tendons. (**B**) Interfibrillar areas were calculated from H&E stained micrographics and shown as percentages (n = 6; **p < 0.01 compared with the healthy group).

To determine how tendon tissues would alter after long-term hyperglycemia, we dissected the Achilles tendons from mice with type 1 DM, the duration of which was at least 1 year, and analyzed the sections using histochemical staining methods. The results indicate that while collagen content was equal to that of the non-diabetic control mice as seen under Masson’s trichrome, the Periodic-Acid Schiff staining demonstrated uneven deposition of glycoproteins and proteoglycans in diabetic tendons (Fig. 7A). Interestingly, diabetic tendons possessed poorly organized collagen fibers, where larger interfibrillar spaces could be seen (Fig. 7A). Through quantitative image analysis by ImageJ software, we confirmed that the average interfibrillar area was significantly larger in diabetic tendons than in healthy tendons (Fig. 7B).

## Discussion

Chronic hyperglycemia, a primary cause of various diabetic complications, serves as a major diagnostic criterion of diabetes mellitus^23^. Diabetic patients with tendinopathy were found to have a longer duration of chronic hyperglycemia than those without tendinopathy^1^. After surgical repair in the rotator cuff, diabetic patients with poorer glycemic control were found to have a higher retear rate^24^. These findings suggest that long-sustained high concentration of blood glucose is associated with diabetic tendinopathy. In the present study, we introduced an *in vitro* diabetic-mimicking culture condition to investigate the effect of high glucose on tenocyte traits. Taking into consideration that hyperinsulinemia also occurs in the early phase of type 2 diabetes, and that insulin might affect cellular glucose metabolism, the effect of insulin on tenocytes in different glucose conditions was also examined. In summary, our results showed that neither glucose nor insulin affected either cell survival or apoptosis. However, tenocyte did have higher glucose uptake and consumed more glucose in high glucose conditions, where insulin further increased the consumption of glucose. We also found that higher glucose consumption was associated with lower AMPK activity in tenocytes, which brought lower expression of tendon-related genes, including transcription factor Egr1 and downstream TGF-β1, Bgn, and Collagen 1. These alterations in gene expression may be involved in the pathomechanism of diabetic tendinopathy.

While ligamental fibroblasts have been shown to exhibit a lowered proliferation rate when cultured for one week in a high glucose condition^25^, studies concerning short-term tenocyte culture within a day suggested no change in both proliferation and apoptosis^18^,^26^. Our results demonstrated that tenocytes maintained similar growth rates between groups up to 14 days. In a previous study, proliferation of human tenocytes was promoted by 30 μg/mL of insulin^27^, whereas that amount of insulin had no effect on our tenocyte culture. In contrast to the culture medium containing 5% FBS supplemented with F-12 used in that study, we used medium containing only 2% FBS in order to imitate the tendon microenvironment, which lacks blood circulation. This less mitogenic condition may have contributed to the mitigation of insulin’s growth effect on tenocyte viability. However, it may also better represent the natural environment of tendons, as we intended, which would suggest that our findings about insulin may be valid. In addition, metabolic activity (NAD(P)H oxidoreductase activity) of cells rather than cell proliferation is measured by the MTT assay. Insulin has also been shown to increase NADH:NAD + ratio and stimulates NAD(P)H oxidase activity in other cells^28^. If a similar effect in tenocytes happened, confounding results of the MTT assay may result in “masking” of an insulin-induced increase in cell proliferation.

Tenocytes were able to uptake glucose more efficiently in HG conditions, although the expression of glucose transporters was unaffected. Insulin did not affect either glucose uptake or expression of Glut4, an insulin-sensitive glucose transporter. This indicates that tenocyte does not rely on Glut4 for glucose uptake, which makes it distinct from muscle cells. According to real-time PCR analysis in a previous study, expression of Glut1 was 2.3-fold as high as Glut4 in Glut4-sensitive muscle cells^29^. In contrast, our results (data not shown) demonstrated that expression of Glut1 was about 15-thousand times higher than Glut4, implying that tenocytes relied mainly on Glut1, the transporter responsible for basal glucose uptake, rather than Glut4. Glut1-reliant cells were shown to be more vulnerable to cellular glucose overload^30^, endorsing our speculation on the adverse effects which hyperglycemia might have on tenocytes.

Cells exposed to higher extracellular glucose concentration tend to consume more glucose and thus inactivate the AMPK signal^21^,^22^. Our glucose consumption analysis confirmed that higher glucose consumption occurred in the HG group, and we additionally found a further increase in consumption when insulin was added to the HG condition. As expected, the more glucose consumed, the lower level of p-AMPKα expressed. It was previously reported that, in muscle cells, AMPK signaling can be regulated by insulin^31^. The expression of p-AMPKα has been shown to possess transcriptional-regulator functions. Activation of AMPK signaling enhanced Egr1 expression, which led to promoted PDK-1 expression in lung cancer cells^32^ and Dusp4 expression in liver cancer cells^33^. In the present study, with higher glucose consumption in diabetic conditions, where AMPK signaling was decreased, expression levels of tendon-related molecules were lowered, including Scx, TGF-β1, type 1 collagen, and Bgn. By administering Compound C to tenocytes in the LG condition, we confirmed that inhibition of AMPK signaling suppressed expression of Egr1 and thus caused down-regulation of those tendon-related genes. Specifically, the regulatory role of Egr1 was examined using siRNA.

Previous studies have shown that tendons from Egr1 gene knockout mice have attenuated mechanical strength, larger interfibrillar spaces under microscope, and suppressed tendon-related genes including Scx, Col1a1, Col1a2, Bgn^10^, and Mkx^13^. In accordance, our histological data demonstrated larger interfibrillar spaces in tendons from DM mice, and all of the aforementioned tendon-related genes were suppressed in Egr1-silenced tenocytes. In addition, inducing overexpression of the Egr1 gene in mesenchymal stem cells (MSCs) was found to promote TGF-β signaling, and implantation of these MSCs into injured rat tendons ameliorated tendon re-formation^10^. In rodents, both TGF-β1 and TGF-β2 expression have been shown to be transcriptionally promoted by Egr1^10^,^34^. Our study showed that knocking-down Egr1 in tenocytes decreased expression of TGF-β1, which was the only TGF-β whose expression decreased in the HG condition.

In the present study, expression of type 1 collagen was significantly reduced in response to both AMPK inhibition and silencing of Egr1. However, the reduction of type 1 collagen caused by either high glucose or insulin was less significant, leading us to speculate that, in addition to AMPK signaling, other pathways were involved in regulating collagen expression under the diabetic conditions. Previous research has found that cardiac fibroblasts in HG conditions expressed higher ERK activity and hence a higher level of Col1a1 than in LG conditions^35^. In tenocytes, glucocorticoid-induced reduction of collagen synthesis was rescued by insulin treatment, which activated both ERK and Akt signals^27^. To our knowledge, whether high glucose affects tenocyte gene expression through activating ERK or Akt has not been reported. In diabetic animal models, Goto-Kakizaki (GK) rats that had type 2 DM for 12 months expressed less type 1 collagen in tendon sections^36^. In contrast, rats with STZ-induced type 1 DM showed increased deposition of type 1 collagen in tendon sections^37^. Therefore, it remains inconclusive how collagen content changes in diabetic tendons. Our finding of a reduction in collagen gene expression suggests that the diabetic environment may have an adverse effect on tenocyte traits, although it did not clarify how collagen content changes in diabetic tendons.

By histological analysis, we demonstrated that the collagen fibers in diabetic tendons were poorly organized without change in collagen content. To attempt to explain this phenomenon, the observed decline in the expression level of growth factors and minor matrix molecules should be emphasized. With overexpression of TGF-β1 cDNA in muscle implants, surgically repaired animal tendon has been shown to exhibit improved mechanical properties as well as better-organized collagen fiber^38^. Previous findings of lower expression levels of TGF-β1 in injured tendons of type-2-DM rats agree with our *in-vitro* result^39^. In addition, a previous study found that TGF-β1 signaling was disrupted in pathological human tendons, which corroborates the crucial role of TGF-β1^17^. Lack of Bgn expression during development has been shown to result in collagen fiber disorganization and calcification in animal tendons^40^. In accordance with our *in-vitro* results, expression of Bgn in injured tendons of type-2-DM rats was lower than in the non-DM group^36^. While we found uneven deposition of glycoproteins in diabetic rat tendons, more *in vivo* evidence is still needed to understand the role of Bgn in diabetic tendons.

While investigating the effect of an *in vitro* diabetic environment on gene expression of rat tenocytes, the present study was limited for several reasons. Firstly, DM, which is a chronic disease, may do more harm on tendon tissues as its duration extends. Our experimental design allowed us to evaluate only short-term effects of *in vitro* diabetic conditions, thus making it hard to connect with *in vivo* results. Secondly, only two factors, glucose and insulin, were analyzed in our study, which cannot fully represent the complex pathophysiological environment in diabetic tendons. Lastly, we examined only the mRNA expression of target genes and not the functional protein level.

**Figure 8:**
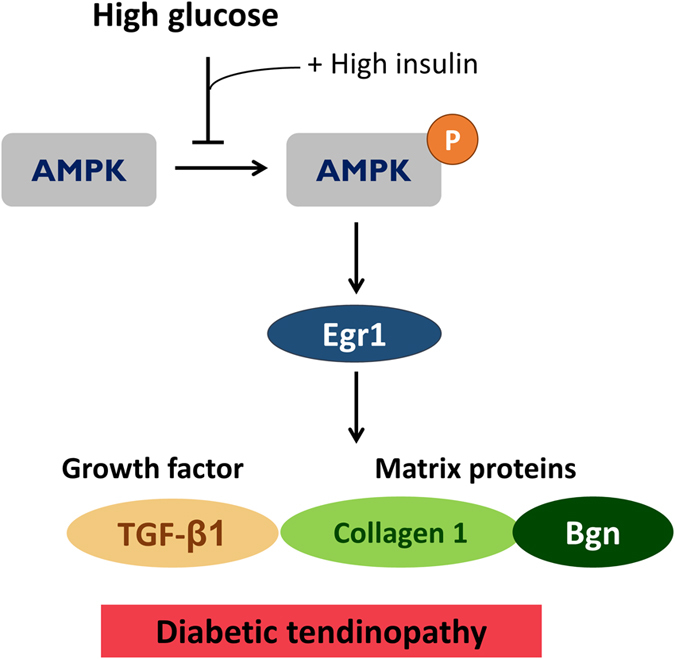
Proposed pathomechanism involved in diabetic tendinopathy.

To sum up, *in vitro* diabetic conditions attenuated the expression of Egr1 by inactivating AMPK signaling, which resulted in the further suppression of downstream tendon-related genes, including TGF-β1, type 1 collagen, and Bgn (Fig. 8). Moreover, diabetic animal tendons were characterized by disorganized structure and uneven matrix deposition, which may be attributed to the aforementioned disruption of tendon-related gene expression. As changes in AMPK-Egr1 molecular signaling may be part of its pathomechanism, therapies activating AMPK are promising for preventing or ameliorating diabetic tendinopathy.

## Research Design and Methods

### Tenocyte culture

The Achilles tendons were isolated from 8-week-old Sprague-Dawley rats. After cutting the tendons into approximately 3-mm^3^ pieces, placing two pieces into a single well of 6-well culture plates (Corning), and maintaining the plates in DMEM with 10% FBS (Gibco) at 37 °C in a humidified atmosphere of 5% CO_2_, primary tenocytes migrated out of the tendon pieces and started expanding at the bottom of the culture plates. The medium was changed every 3 days, and cells were trypsinized and subcultured onto 100-mm dishes when they reached subconfluency at about day 10–14. Cells from passage 3 to 10, which were normal fibroblast-shaped, were used for all the following experiments (Supplementary Fig. S1). All study procedures received approval of the Institutional Animal Care and Use Committee of National Taiwan University College of Medicine and College of Public Health (IACUC Approval No: 20150362), and the Guide for the Care and Use of Laboratory Animals (Chinese-Taipei Society of Laboratory Animal Science).

### Cell viability

The MTT (3-[4,5-Dimethylthiazol-2-yl]-2,5-diphenyltetrazolium bromide) assay was used to assess tenocyte viability. Tenocytes were seeded in 96-well plates and then incubated in low (5.5 mM) or high (25 mM) glucose medium, with (30 μg/mL)^27^ or without insulin. At each time point, medium was replaced with PBS containing 2.5 mg/mL MTT, and the plates were incubated at 37 °C for 5 hours. Afterwards, MTT solution was removed, and 100 μL DMSO was added to each well to dissolve the purple crystals. Absorption at 570 nm was detected using an ELISA reader.

### Cell apoptosis

To detect mitochondrial membrane potential, an indicator of cell apoptosis, the JC-1 assay kit (GeneCopoeia) was used according to the manufacturer’s instructions. Briefly, after tenocytes in 96-well plates were incubated in low- or high-glucose medium, with or without insulin, for 14 days, media were replaced with PBS and cationic JC-1 dye was added. After incubation at 37 °C for 30 minutes, JC-1 solution was replaced with fresh PBS, and red and green fluorescence in each well were observed. The cellular image was acquired with an Olympus IX71 fluorescence microscope combined with an Olympus DP72 camera and cellSens Standard 1.14 software (Olympus, Germany).

### 2-NBDG glucose uptake assay

2-NBDG (2-(N-(7-nitrobenz-2-oxa-1,3-diazol-4-yl)amino)-2-deoxyglucose) is a fluorescent analogue of glucose, which allows observation of glucose uptake in each cell. Tenocytes in 96-well plates were incubated in low- or high-glucose medium, with or without insulin. At each time point, the medium was replaced with fresh medium containing either 45 μM (low glucose) or 180 μM (high glucose) 2-NBDG, and was incubated at 37 °C for 45 minutes. Then, the 2-NBDG solution was replaced with fresh medium without 2-NBDG. After incubation at 37 °C for an additional 5 minutes to allow the non-phosphorylated 2-NBDG to move out of the cells, the medium was replaced with PBS, and green fluorescence in each well was observed. The cellular image was acquired with the fluorescence microscope and camera (Olympus).

### Measurement of glucose in culture medium

Tenocytes in 6-well plates were incubated in low- or high-glucose medium, with or without insulin, and the incubated medium was collected at day 7. The GM300 blood glucose monitor (Bionime) was first calibrated using standard solutions containing a series of glucose concentrations, including low glucose, high glucose, and mixing low and high glucose of fresh DMEM. The glucose concentration of the medium was calculated using a calibration curve with an R square of 0.9995.

### RNA extraction and real-time quantitative PCR analysis

Total RNA was extracted from cells on 6-well plates using TRI Reagent and Direct-zol^TM^ RNA Kit (Zymo Research), and 500 ng RNA was reverse transcribed into cDNA with Tetro cDNA Synthesis Kit (Bioline). Quantitative real-time PCR was performed using the ABI 7900 system (Applied Biosystems) with a SensiFAST^TM^ SYBR® Hi-ROX Kit (Bioline), where each reaction mixture contained 50 ng of cDNA. Sequences of the primer pairs used were: HPRT1, forward primer (5′-AAGCTTGCTGGTGAAAAGGA-3′), reverse primer (5′-CCGCTGTCTTTTAGGCTTTG-3′); GAPDH, forward primer (5′-GTGGACCTCATGGCCTACAT-3′), reverse primer (5′-GGATGGAATTGTGAGGGAGA-3′); Scx, forward primer (5′-TTGAGCAAAGACCGTGACAG-3′), reverse primer (5′-CTGTGCTCAGATCAGGTCCA-3′); Mkx, forward primer (5′-CTCCGGTTTCGATTGAGAAG-3′), reverse primer (5′-AGTTGTTCACGGCTCTTCGT-3′); Egr1, forward primer (5′-TTATCCCAGCCAAACTACCC-3′), reverse primer (5′-CAGAGGAAGACGATGAAGCA-3′); Col1a1, forward primer (5′-GGAAGAGCGGAGAGTACTGG-3′), reverse primer (5′-CATGCTCTCTCCAAACCAGA-3′); Col1a2, forward primer (5′-TTGAATACAACGCAGAAGGG-3′), reverse primer (5′-CCTGCAGAATGACAGCCTTA-3′); Dcn, forward primer (5′-TTCACCCACACACCTTCAGA-3′), reverse primer (5′-GCATCCGCGCTATACTTGA-3′); Tnmd, forward primer (5′-CCTCAGCAGTGGTCTCTCAG-3′), reverse primer (5′-AAGGCCAGGATACCAAACAC-3′); Tnc, forward primer (5′-TGCCATGAAGGGATTTGAAGA-3′), reverse primer (5′-GGCTGTCAGAAGGCCAGATG-3′); Bgn, forward primer (5′-AGCTGTACAGGCTGGGCTTA-3′), reverse primer (5′-CACCTTGGTGATGTTGTTGG-3′); Sox9, forward primer (5′-CTGCGACCTCAGAAGGAAAG-3′), reverse primer (5′-CGCTGGTATTCAGGGAGGTA-3′); Runx2, forward primer (5′-AAGTGCGGTGCAAACTTTCT-3′), reverse primer (5′-AGGCTGTTTGACGCCATAGT-3′); PPARγ, forward primer (5′-CATTTTTCAAGGGTGCCAGT-3′), reverse primer (5′-GAGGCCAGCATGGTGTAGAT-3′); Tgfb1, forward primer (5′-ATACGCCTGAGTGGCTGTCT-3′), reverse primer (5′-TGGGACTGATCCCATTGATT-3′); Tgfb2, forward primer (5′-CTGGAACCACTGACCATCCT-3′), reverse primer (5′-AACTCCCTCACGTCACGAAC-3′); Tgfb3, forward primer (5′-GCGTCTCAAGAAGCAGAAGG-3′), reverse primer (5′-GCAGTTCTCCTCCAAGTTGC-3′); Glut1, forward primer (5′-GCCTGAGACCAGTTGAAAGC-3′), reverse primer (5′-GAGTGTCCGTGTCTTCAGCA-3′); and Glut4, forward primer (5′-GTCTTCACGTTGGTCTCGGT-3′), reverse primer (5′-CTCAAAGAAGGCCACAAAGC-3′). The PCR reaction began with a 2-minute polymerase activation, followed by 40 cycles of 3-step amplification—denaturation at 95 °C for 5 seconds, annealing at 60 °C for 10 seconds, and extension at 72 °C for 10 seconds. Afterwards, melting curves of amplified PCR products were analyzed from 60 °C to 95 °C. The relative gene expression level was calculated by the 2^(−ΔΔCT)^ method, with HPRT1 and GAPDH as internal controls.

### Western blotting

Total soluble protein was collected from cells on 6-well plates using RIPA buffer (Millipore). The SDS-PAGE was performed with a 10% gel, and then the proteins were transferred onto PVDF membranes. Following blocking of the membrane by TBST buffer containing 0.05% Tween 20 and 5% BSA for 1.5 hr at room temperature, the membrane was incubated in the buffer with primary antibody targeting phospho-AMPKα or β-Actin at 4 °C overnight and then with HRP-conjugated secondary antibody. After adding Chemiluminescent HRP Substrate (Millipore) to the membrane, the luminescence images were acquired by ImageQuant LAS 4000 and analyzed by ImageQuantTM TL8.1 software (GE Healthcare, Uppsala, Sweden).

### Small interference RNA transfection

Egr1 or Mkx genes were knocked down using specific siRNAs, which were transfected into tenocytes by the GenMute^TM^ siRNA Transfection Reagent (SignaGen®). Sequences of the siRNAs used were: FAM-labeled negative control, 5′-UUCUCCGAACGUGUCACGUdTdT-3′; siNCtrl, 5′-UUCUCCGAACGUGUCACGUdTdT-3′; siMkx, 5′-GCUGUGUCGUAGAGAUUUAdTdT-3′ and 5′-AGUUGGGCCUUAAGAAUAAdTdT-3′ that were simultaneously transfected; and siEgr1, 5′-GGACAAGAAAGCAGACAAAdTdT-3′ and 5′-GAUGAACGCAAGAGGCAUAdTdT-3′ that were simultaneously transfected. Transfection efficiency was evaluated using FAM-labeled siRNA as an indicator. After 48 hours, knockdown efficiency was confirmed by qRT-PCR, and then all the downstream genes were analyzed.

### *In vivo* mice diabetic model

All procedures involving experimental animals were performed in accordance with the guidelines for animal care of National Taiwan University and complied with the “Guide for the Care and Use of Laboratory Animals” (NIH publication No. 86–23, revised 1985). The DM mice were kindly provided by Dr. Hong-Wei Chang, Department of Internal Medicine, National Taiwan University Hospital. To induce long-term type 1 diabetes, C57BL/6 mice (8-weeks old) were injected intraperitoneally with high-dose streptozotocin (STZ; 170 mg/kg; Sigma-Aldrich)^41^. After injection, blood glucose levels were monitored once a week and kept no lower than 450 mg/dL for one year. In the non-DM group, mice were injected intraperitoneally with saline.

### Histochemistry

One year after DM induction, mice were sacrificed by CO_2_ asphyxiation, and the Achilles tendons were carefully excised and collected. Tissue samples were then fixed in 4% paraformaldehyde, embedded in paraffin, and dissected. For tissue morphological examination, sections were stained with hematoxylin-eosin, and 6 separate fields from 4 specimens were analyzed by ImageJ software. For collagen and glycoprotein content observation, sections were stained with Masson’s trichrome and periodic acid-Schiff reagent, respectively.

### Statistical analysis

All the data are expressed as mean ± SD. To evaluate the differences between two groups, an independent t test was performed, and for comparisons between three or four groups, one-way ANOVA was performed. Tukey’s post hoc test was used to examine the glucose effect or insulin effect, following a significant ANOVA. Differences with a p-value less than 0.05 were considered to be significant.

## Additional Information

**How to cite this article:** Wu, Y.-F. *et al*. High glucose alters tendon homeostasis through downregulation of the AMPK/Egr1 pathway. *Sci. Rep.* **7**, 44199; doi: 10.1038/srep44199 (2017).

**Publisher's note:** Springer Nature remains neutral with regard to jurisdictional claims in published maps and institutional affiliations.

## Supplementary Material

Supplementary Figure
