# Supplementary material for: High glucose alters tendon homeostasis through downregulation of the AMPK/Egr1 pathway
Source: Sci Rep. 2017 Mar 7;7:44199. doi: 10.1038/srep44199 (PMC5339827; doi:10.1038/srep44199)
Supplement: Supplementary Figure [file srep44199-s1.pdf]

Supplementary Figure

High glucose alters tendon homeostasis through downregulation of the AMPK/Egr1 pathway

Yu-Fu Wu[1], Hsing-Kuo Wang[1,2], Hong-Wei Chang[3], Jingyu Sun[4], Jui-Sheng Sun[5,6], Yuan-Hung Chao[1,2,7*]

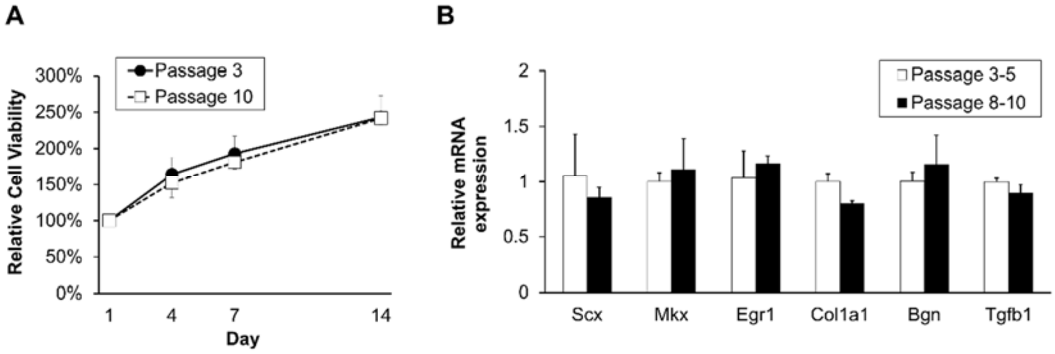

Supplementary Fig. S1. Effects of passages on the phenotype of the rat tenocytes. (A) The growth rate was similar between passage 3 and passage 10. (B) Expression of tendon-related genes was stable between passages 3-5 and passages 8-10. (n=6)

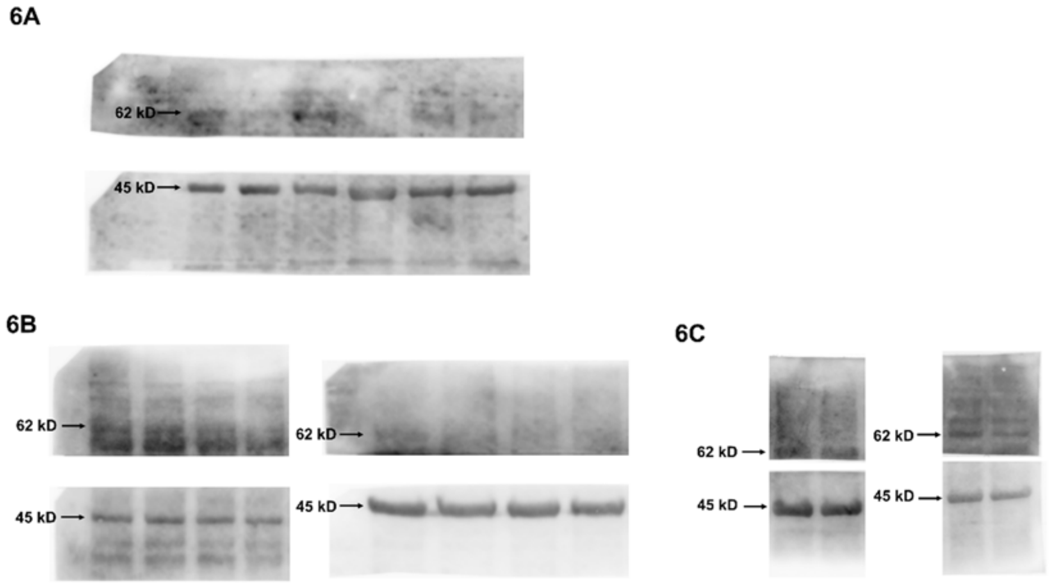

Supplementary Fig. S2. Uncropped Western blots related to Fig. 6.
